# The Formation of γ-Valerolactone from Renewable Levulinic Acid over Ni-Cu Fly Ash Zeolite Catalysts

**DOI:** 10.3390/molecules29235753

**Published:** 2024-12-05

**Authors:** Margarita Popova, Silviya Boycheva, Ivan Dimitrov, Momtchil Dimitrov, Daniela Kovacheva, Daniela Karashanova, Nikolay Velinov, Genoveva Atanasova, Agnes Szegedi

**Affiliations:** 1 Institute of Organic Chemistry with Centre of Phytochemistry, Bulgarian Academy of Sciences, 1113 Sofia, Bulgaria; ivan.dimitrov@orgchm.bas.bg (I.D.); momtchil.dimitrov@orgchm.bas.bg (M.D.); 2 Department of Thermal and Nuclear Power Engineering, Technical University of Sofia, 8 Kl. Ohridsky Blvd., 1000 Sofia, Bulgaria; sboycheva@tu-sofia.bg; 3 Institute of General and Inorganic Chemistry, Bulgarian Academy of Sciences, 1113 Sofia, Bulgaria; didka@svr.igic.bas.bg (D.K.); genoveva@svr.igic.bas.bg (G.A.); 4 Institute of Optical Materials and Technologies, Bulgarian Academy of Sciences, 1113 Sofia, Bulgaria; dkarashanova@yahoo.com; 5 Institute of Catalysis, Bulgarian Academy of Sciences, 1113 Sofia, Bulgaria; nikivelinov@ic.bas.bg; 6 HUN-REN Research Centre for Natural Sciences, Institute of Materials and Environmental Chemistry, Magyar Tudósok Krt. 2, 1117 Budapest, Hungary

**Keywords:** valorization of coal fly ash, zeolites, lignocellulosic biomass, levulinic acid, γ-valerolactone

## Abstract

Zeolites with different structures (P1, sodalite, and X) were synthesized from coal fly ash by applying ultrasonically assisted hydrothermal and fusion–hydrothermal synthesis. Bimetallic catalysts, containing 5 wt.% Ni and 2.5 wt.% Cu, supported on the zeolites, were prepared by a post-synthesis incipient wetness impregnation method. The catalysts were characterized by X-ray powder diffraction (XRPD), N_2_ physisorption, transmission electron microscopy (TEM), Mössbauer and X-ray photoelectron spectroscopies (XPS), and H_2_–temperature-programmed reduction (H_2_-TPR) analyses. The XRPD results showed that crystalline Cu^0^ and Ni_x_Cu_y_ intermetallic nanoparticles were formed in the reduced catalysts. The presence of the intermetallic phase affected the reducibility of the nickel by shifting it to a lower temperature, as confirmed by the H_2_-TPR curves. Based on the Mössbauer spectroscopic results, it was established that the iron contamination of the coal fly ash zeolites (CFAZs) was distributed in ionic positions of the zeolite lattice and as a finely dispersed iron oxide phase on the external surface of the supports. The formation of the NiFe alloy, not detectable by XRPD, was also evidenced on the impregnated samples. The catalysts were studied in the upgrading of levulinic acid (LA), derived from lignocellulosic biomass, to γ-valerolactone (GVL), in a batch reactor under 30 bar H_2_ pressure at 150 and 200 °C, applying water as a solvent. The NiCu/SOD and NiCu/X catalysts showed total LA conversion and a high GVL yield (>75%) at a reaction temperature of 200 °C. It was found that the textural parameters of the catalysts have less influence on the catalytic activity, but rather the stable dispersion of metals during the reaction. The characterization of the spent catalyst found the rearrangement of the support structure. The high LA conversion and GVL yield can be attributed to the weak acidic character of the support and the moderate hydrogenation activity of the Ni-Cu sites with high dispersion.

## 1. Introduction

The utilization of lignocellulosic biomass as an alternative to fossil fuel sources for energy supply offers a potential solution to the depletion of fossil fuel resources and the negative ecological impacts associated with their use [1,2,3]. However, direct biomass combustion is not a sustainable approach to energy production due to several challenges. These include the high energy costs of fuel pretreatment, large fuel volumes, the slagging of furnace chambers due to the increased content of low-melting alkali oxides in the ash residue, and the elevated risk of cyclic hydrocarbon emissions during incomplete combustion [4,5]. Consequently, alternative methods for converting biomass into liquid fuels are being explored. Lignocellulosic biomass can be hydrolyzed into a mixture of cellulose, hemicellulose, and lignin [6]. The subsequent transformation of hemicellulose and cellulose to C5 and C6 monosaccharides is a critical step in biomass valorization. Among the products derived from biomass, levulinic acid (LA) stands out as a key platform molecule. LA serves as a precursor for the production of valuable products, such as biofuels, pharmaceutical intermediates, solvents, etc. [7,8,9,10].

Most of the processes for the LA conversion are catalytic, making the synthesis of efficient heterogeneous catalysts a matter of primary importance. However, many of the most effective catalysts proposed for this process are based on costly and scarce precious metals [11]. Ni-, Co-, and Cu-based catalysts exhibit great potential in hydrogenation reactions and present an appropriate alternative of noble-metal-based catalysts (Ru, Ir, and Pd) [10,12,13,14]. Heterogeneous catalysts offer the advantages of easy recovery and recycling [15]. However, their applications are often limited by drawbacks such as metal agglomeration, the inaccessibility of active centers to the reactants, and the leaching of the active phase under reaction conditions [16,17]. The development of efficient catalysts is an important step toward the sustainable production of GVL by the hydrogenation of LA.

During the treatment of levulinic acid with hydrogen, a number of parallel and sequential reactions take place, including hydrogenation–dehydrogenation, hydrogenolysis, dehydration, esterification, decarboxylation, etc. The selective catalytic conversion of levulinic acid in each of these processes requires a thorough understanding of the reaction mechanisms to guide the reaction in the desired direction. Nickel-based catalysts, supported on γ-Al_2_O_3_, silica, MgO, or H-ZSM-5, are known to be active in the hydrogenation reaction [18]. Ni and Cu, supported on γ-Al_2_O_3_, acted as highly selective catalysts to produce 2-methyltetrahydrofuran with 80% selectivity when propanol was used as a solvent at 250 °C and at 40 bar [19]. Similarly, a Ni-Cu-modified H-ZSM-5 catalyst, prepared by impregnation, exhibited a superior catalytic performance compared to that prepared via ion exchange [20]. It achieved a 70% LA conversion with 62% selectivity to GVL formation at 300 °C and a GHSV = 8.166 mL s^−1^ g cat^−1^. Another example is the ZrO_2_/SBA-15 catalyst, with a ∼95% yield of GVL from LA at 150 °C under 1.0 MPa argon after a 3 h reaction [21]. The reaction medium for the LA conversion typically involves water or organic solvents such as methanol, ethanol, 1-butanol, 2-butanol, and 1,4-dioxane. In the presence of secondary alcohols, catalytic hydrogen transfer reactions can occur. However, using them as a hydrogen source can reduce hydrogenation selectivity due to the competing esterification reaction.

The choice of carrier materials significantly impacts the performance of catalysts. It has been established that the catalytic activity and the selectivity of several heterogeneous catalysts can be optimized by controlling the structure, surface characteristics and porosity of the supports. This, in turn, regulates the transport of reactants and enhances the activity of the catalysts [22,23]. Zeolites are among the preferred catalytic carriers due to their high specific surface area, well-defined micropore structure with molecular-sized pores, high adsorption capacity, the presence of acid centers in the zeolite framework, and the ability to host uniformly distributed, additionally created metal active sites. Furthermore, the synergistic interaction between the zeolite carrier and the active catalytic centers adds to their effectiveness. The flexibility to tune the pore structure, crystal morphology, and the incorporation of a variety of active sites through modification and functionalization allows for the improvement of the activity of the zeolite catalyst [24,25]. Zeolites obtained from coal fly ash (CFA) present a promising alternative to commercial zeolites, offering not only a cost-effective option but also addressing the environmental concerns associated with CFA deposition. Various types of zeolites can be easily synthesized from CFA through alkaline hydrothermal activation, with or without a pre-fusion stage of the reaction mixtures. This process allows for tailoring the synthesis conditions, including temperature, treatment duration, the type of alkaline activator, and the use of additives, etc., to obtain the desired zeolite properties [26,27].

Coal fly ash zeolites (CFAZs) are promising catalytic carriers and catalytic systems in their own right, due to their adequate specific surface area, the potential for post-synthesis modification, and the incorporation of metal and metal oxide particles into the zeolite framework from the raw CFA [8,10,12]. A key advantage of CFAZs over pure synthetic zeolites is their mixed micro-mesoporous structure, which facilitates the mass transfer within the material [10,12]. In our previous studies, nanocrystalline, micro-mesoporous Na-X zeolite was successfully synthesized by using lignite CFA and investigated as both a carbon dioxide adsorbent and a catalyst for the oxidation of volatile organic compounds [8,9,10,12]. The high catalytic activity of the CFAZ catalysts was achieved by post-synthesis modification with transition metal oxides.

In the present study, in addition to Na-X, other zeolite phases with potential catalytic applications were synthesized and investigated. This study focuses on the preparation of Ni- and Cu-functionalized coal fly ash zeolites by post-synthesis impregnation. The catalysts were studied during the hydrogenation of levulinic acid to γ-valerolactone. Notably, the presence of the iron phase in the prepared fly ash zeolites was found to positively influence the nickel dispersion, resulting in enhanced catalytic activity.

## 2. Results and Discussion

In this study CFAZs of faujasite (X), hydrosodalite (SOD), and gismondine (P1) types were synthesized from CFA_AES_ and CFA_DM_ raw materials using ultrasonically assisted hydrothermal and double-stage fusion–hydrothermal synthesis by varying the temperature and the duration of hydrothermal activation. The synthesis procedures are described in the Experimental Section and summarized in Table 1. For the conversion of the CFA to zeolites, two experimental techniques were applied. The first was a two-step synthesis process involving the alkaline fusion of solid reaction mixtures of CFA and an alkaline activator (NaOH), followed by the preparation of aqueous slurries from the milled and cooled charges and subsequent hydrothermal synthesis. The second technique was a one-step process consisting only of the hydrothermal activation of CFA alkaline slurries. Prior to hydrothermal stage in both experimental techniques, the ultrasonic homogenization of the reaction slurries was applied. The investigations revealed that the zeolitization of CFA proceeds through the following main stages: the dissolution of the aluminosilicates from the CFA composition in the alkaline solution, the formation of silica and alumina hydrogels, and the subsequent heterogeneous crystallization of the hydrogel to the zeolite phase on the solid particles of alkaline-resistant components in the CFA [28]. The two experimental approaches offer distinct advantages in the synthesis of zeolite phases. The inclusion of a preliminary fusion stage achieves a higher degree of the zeolitization of the raw CFA. During this high thermal treatment, the chemically inert crystalline phases in the CFA, such as quartz, mullite, and anorthite, are converted into water-soluble alkaline silicates and aluminates, which actively participate in the zeolitization process. Conversely, in the one-step hydrothermal activation, these inert phases remain stable and are incorporated into the final zeolite product.

The raw CFA contains iron components, predominantly iron oxide spinel phases, such as magnetite and maghemite. During ultrasonic treatment, these nanoparticles are dispersed, and ionic iron is dissolved. They can be included in the aluminosilicate composition, providing ferro and paramagnetic iron oxide nanoparticles and Fe^2+^/Fe^3+^ ions in the zeolite lattice. During alkaline melting, the spinel iron oxides oxidize almost completely to hematite, which is dispersed in the zeolite as hematite nanoparticles and, additionally, supplies Fe^3+^ ions to the zeolite lattice. Extra-framework Fe^3+^ ions can act as Lewis acid sites, thus improving the catalytic efficiency [29]. The iron oxide phase transition during CFA thermal treatment was previously studied [30]. Although the two-stage synthesis has clear advantages in the crystallinity of the zeolite, a variety of zeolitic phases can be produced in one-stage hydrothermal synthesis from the same CFA composition without any additives, by varying the alkalinity, temperature, and time parameters. It can be associated with the slower dissolution of the aluminosilicate components in the alkaline solution and the possibility of changing the Si/Al ratio within a wider range in the reaction mixture [31,32,33,34]. The thermodynamic sequence for the crystallization of different zeolite phases via the hydrothermal activation of a distinct CFA composition was studied in our previous work [35]. In both synthesis procedures, ultrasonic treatment was applied for reaction mixture homogenization. It was found that it shortens the time of CFA processing to zeolite, accelerates the secondary nucleation rate, ensures a nanocrystalline morphology of the CFAZs, and leads to a uniform distribution of the iron oxide components incorporated into the products [36]. Ultrasonic treatment increases the solubility of the alkali-resistant phases of the raw CFA and improves the zeolite quality also in the one-step hydrothermal activation.

The elemental composition of initial zeolite impurities was determined by an ICP–OES analysis (Table S1). Some impurities, such as Cu, Zn, Cr, Ni, Ba, Li, B, Co, Pb, Cd, and Bi were detected at the ppm level, whereas iron content was measured around 7.8–7.9 wt.% in all the zeolites.

**Figure 1:**
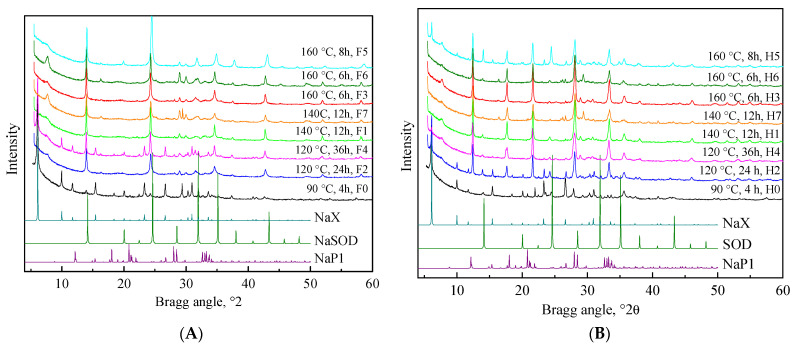
(**A**). XRPD patterns of the CFAZs obtained by ultrasonically assisted double-stage fusion-hydrothermal synthesis. (**B**). XRPD patterns of the CFAZs obtained by ultrasonically assisted one-step hydrothermal synthesis.

The XRD patterns of the materials obtained are presented in Figure 1. The synthesis conditions have a strong impact on the properties of the prepared zeolites. By applying an NaOH/CFA/NaCl ratio of 5/8/2, a fusion temperature of 550 °C, 15 min of ultrasonic treatment, and a hydrothermal treatment at 160 °C for 8 h, sodalite zeolite was prepared (F1–F7). When the synthesis procedure was performed without the fusion step (H1–H7), the most appropriate conditions for zeolite synthesis, containing predominantly P1 type, were an NaOH/CFA reaction mixture ratio of 5/8, 30 min of ultrasonication, and 6 h of hydrothermal treatment at 160 °C.

Based on our previous experience, the appropriate conditions for X zeolite (F0) synthesis were an NaOH/CFA ratio of 5/10, a fusion temperature of 550 °C, 15 min of ultrasonic treatment, and hydrothermal treatment at 90 °C for 4 h.

Based on these results, three samples presenting different zeolites (X, SOD, and P1) were selected for the further development of the catalysts by impregnation with metal salt precursors. H3, F5, and F0 were selected because they contain P1, SOD, and X zeolites, respectively, with the lowest amount of impurities. The selected zeolite X contains 3% zeolite A, whereas the selected zeolites SOD and P1 contain 15% and 25% tobermorite, a calcium silicate with a composition of Ca_5_(OH)_2_Si_6_O_6_·4H_2_O.

**Figure 2:**
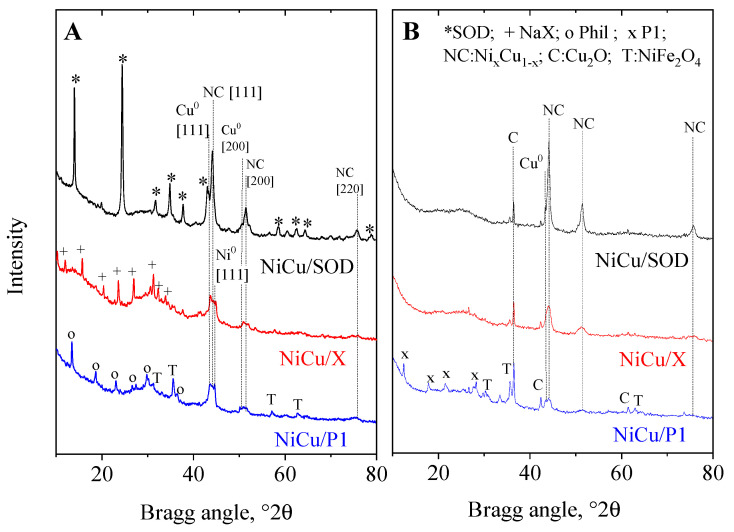
XRPD patterns of the initial, reduced catalysts (**A**), and their spent varieties (**B**).

The XRPD patterns of the reduced and the spent catalysts are shown in Figure 2, and the crystalline phases, with their crystallite size, are summarized in Table 2. All the catalysts showed crystalline zeolite phases of the corresponding types, except for the NiCuP1 sample, where the zeolite reflections indicated phillipsite. This structural transformation was also reported by Huo et al. [37,38], regarding a heat treatment between 400–800 °C. The original cubic type, P1, was transformed into tetragonal symmetry upon dehydration at 100 °C, then to phillipsite over 400 °C, and to nepheline at 1000 °C [37,38].

In all the reduced catalysts, a Ni_x_Cu_y_ intermetallic phase and metallic copper could be determined. The unit cell size of the intermetallic phase, in most cases, was 3.554 nm, indicating an x = 0.67 value based on Vegard’s law [39], showing the formation of a nickel-rich alloy. The amount and the crystallite size of the NiCu phase were the largest on the sodalite catalyst, while the other two zeolites showed similar, but smaller amounts and sizes of nanoparticles, around 12 nm. A small amount of metallic copper also appeared in the patterns, with crystallite sizes ranging from 26–120 nm. The finest dispersion of copper was attained on the P1 zeolite. As a consequence of Cu^0^ formation, metallic nickel could be also observed in the X and P1 samples. The formation of a spinel-type ferrite (NiFe_2_O_4_, trevorite) was also detected in the latter sample, indicating that the iron content of the catalysts can interact with the impregnating metals, which can influence catalytic activity.

**Figure 3:**
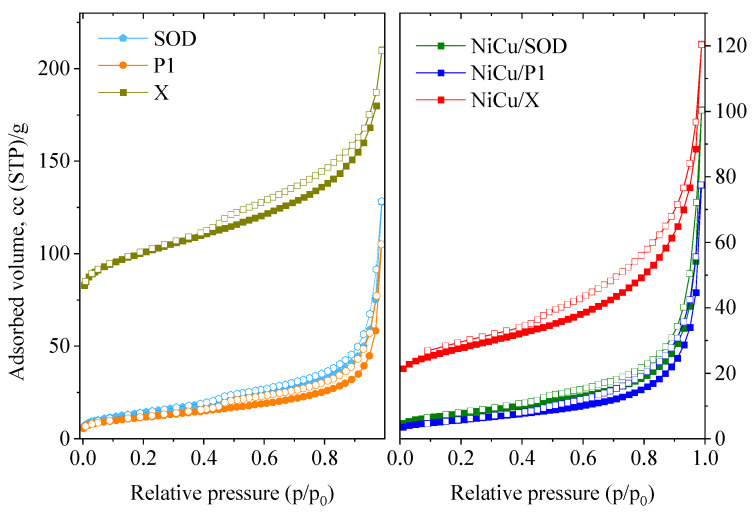
N_2_ physisorption isotherms of the studied samples (solid color—adsorption branch; hollow color—desorption branch).

Nitrogen physisorption isotherms are shown in Figure 3, and the calculated surface parameters are listed in Table 3. The initial zeolites show type I isotherms typical of zeolites, with a steep rise at low relative pressures. The samples also displayed some mesoporous characteristics, with a type H3 hysteresis loop, typical of agglomerated nanoparticles with wide pore size distributions. Comparing the catalysts, zeolite X exhibited a much higher microporosity than the other two. The relatively low micropore volume of the SOD and P1 samples can be explained by the narrower pores of them and by the occupation of cationic positions in zeolites of metal ions originating from CFA, making the pores inaccessible to nitrogen. According to the IZA online database, the pore dimension of SOD for diffusion is 2.59 Å, whereas for P1, it is somewhat bigger at 3.32 Å. The accessible volume of SOD is 0.0%, and it is 9.38% for P1. Through nitrogen adsorption, practically only the external surface of the zeolite and the mesopore volume can be measured. Metal impregnation further reduced the surface area, most notably in the case of the X zeolite. The deposited transition metal oxides blocked some mesopores, and also a part of the micropores, by ion exchange, in the case of zeolite X.

**Figure 4:**
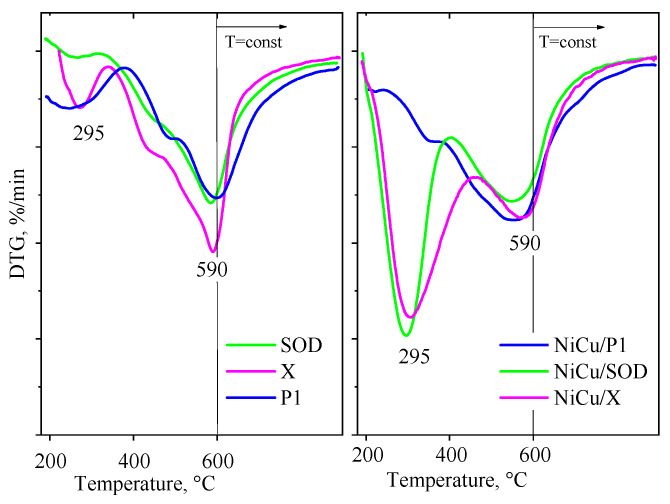
TPR-TG profiles of the catalysts studied.

The TPR-DTG curves for the initial SOD, P1 and X zeolites, along with their NiCu-modified analogues are presented in Figure 4. The parent catalysts display an intense peak at 590 °C, which corresponds to the reduction of cationic Fe^3+^/Fe^2+^ species and, simultaneously, to the reduction of finely dispersed Fe_2_O_3_ nanoparticles to the metallic state. The iron content of zeolite originates from the contamination of the CFA that was used as a raw material for their synthesis. The reduction peak at elevated temperatures is usually associated with the more difficult reduction of ionic species situated in the zeolite lattice [40].

The TPR profiles of the NiCu-modified zeolites are characterized by two intense reduction peaks at 295 °C and 590 °C. The high temperature peak is related to the reduction of iron species, as was shown before for the initial zeolites. The lower temperature step is primarily attributed to the reduction of CuO nanoparticles with variable dispersion on the external surface of the zeolite. NiO reduction takes place at higher temperatures, between the reduction of copper and iron oxides.

According to the literature, separate NiO species are typically reduced between 400 and 500 °C, or even above 500 °C, when they strongly interact with the support [41]. The drastic decrease in the reduction temperature of the NiCu/X and SOD zeolites may be due to the formation of an intermetallic compound, as suggested by the XRD results. Additionally, Cu-catalyzed H_2_ activation likely promotes the low temperature reduction of the NiO species [41]. The reduction profile of NiCu on the P1 zeolite shows a different character, with a smaller low-temperature peak. The NiCu species in this sample are reduced in the same regime as the iron species, with a lower extent of reduction (approximately 42%). This contrasts with the other two zeolites, where the total reduction to the metallic state is achieved up to 600 °C. The lower reducibility of the P1-supported catalyst could be due to structural features, such as the crystallographic transformation resulting in the encapsulation of metal oxide species, hindering their reducibility.

**Figure 5:**
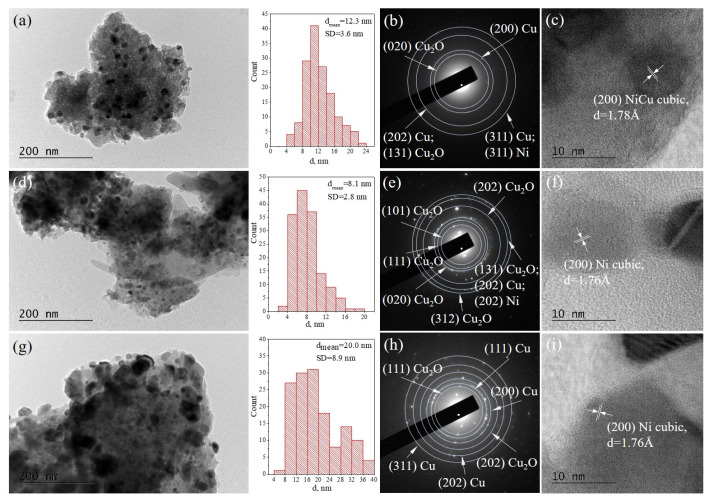
Bright Field TEM micrographs, the size distribution of the metal/alloy nanoparticles, and the corresponding SAED patterns and HRTEM images of the reduced samples: (**a**–**c**) NiCu/X, (**d**–**f**) NiCu/P1, and (**g**–**i**) NiCu/SOD.

The morphology and the microstructure of the reduced catalysts—NiCu/X, NiCu/P1, and NiCu/SOD—were studied by transmission electron microscopy (TEM) in Bright Field (BF) mode. Selected area electron diffraction (SAED) was applied to identify the phase composition. The BF TEM micrographs and SAED patterns are presented in Figure 5. Metallic Cu (cubic, a = 3.63689 Å, COD Entry #96-151-2505) and Cu_2_O (cubic, a = 4.26850 Å, Crystallography Open Database (COD) Entry #96-900-7498) were identified in the diffraction patterns, while metallic Ni (cubic, a = 3.52414 Å, COD Entry #96-151-2527) and intermetallic NiCu nanoparticles (cubic, a = 3.56360 Å, COD Entry #96-152-4233) were visualized by High Resolution TEM micrographs.

The crystalline phases, identified by the TEM analysis, are in accordance with the XRD and XPS results. Histograms based on the statistical analysis reveal that the nanoparticle size distributions for all the catalysts coincide well with the values determined by XRD. The results are presented in Table 2.

**Figure 6:**
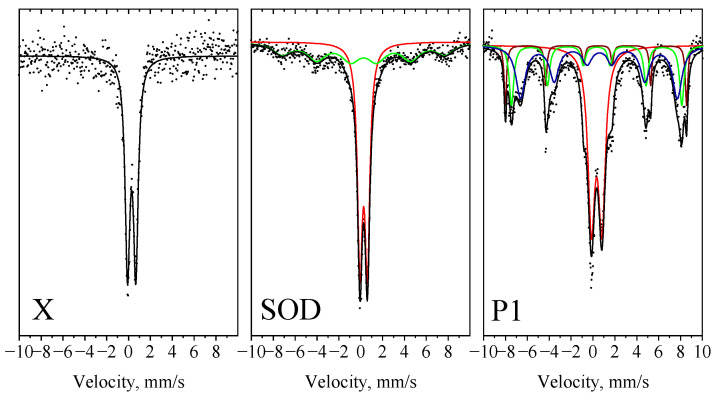
Mössbauer spectra of the initial zeolites.

To study the effect of iron content in the catalysts, Mössbauer spectroscopy was applied to determine the siting, the oxidation state, and the coordination of iron ions in the zeolite matrix. The results are presented in Figure 6, and the parameters, such as isomer shift (*δ*), quadruple splitting (Δ (2*ε*)), hyperfine magnetic field (*B_hf_*), line width (*Γ_exp_*), and the relative weight of each component (G), are summarized in Table 4.

In the Mössbauer spectrum of sample X, a doublet characteristic of the Fe^3+^ phase was observed. In the SOD zeolite, a similar doublet was accompanied by a very broad sextet. Due to the lack of clear lines of the sextet component, it is difficult to determine exactly the crystal phase it belongs to. It may be a ferrite phase (e.g., maghemite, γ-Fe_2_O_3_) with a defective spinel structure due to the small crystallite size and/or due to substituting cations. For the P1 zeolite, three sextets were observed in addition to the doublet. The calculated parameters suggest that the sextets correspond to the hematite and magnetite phases, as presented in Table 4.

**Figure 7:**
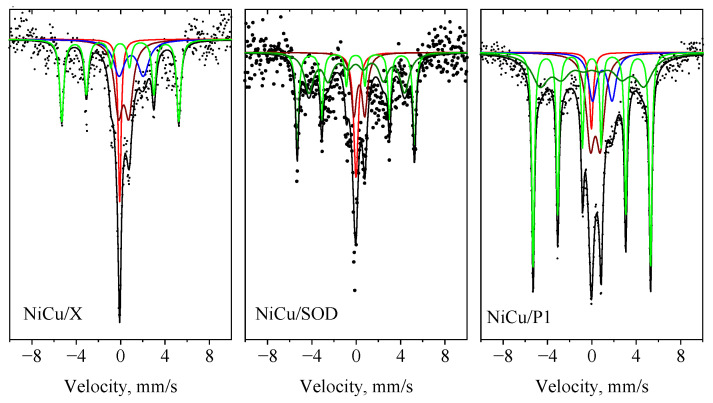
Mössbauer spectra of the nickel- and copper-modified and 550 °C reduced samples.

The Mössbauer spectra of the nickel- and copper-modified, reduced samples are shown in Figure 7, and the Mössbauer parameters determined are summarized in Table 5. These spectra reveal several additional components compared to the initial zeolites. Doublets corresponding to ionic iron species are also present, as a hardly reducible lattice ion in the zeolites (Db1-Fe^3+^). Additionally, Db2 doublets with an isomeric shift of δ = 0.95 and high quadrupole splitting values (1.79–2.88) are characteristic of Fe^2+^ species in the X and P1 zeolites, indicating a partial reduction of these ionic species in 30–40%. As a result of the reduction process, a singlet and two types of sextets were detected, identified as metallic iron or an iron–nickel alloy, which are the predominant phases in the samples. In zeolite X, about half of the iron content exists in the zero-valent state, whereas this value is much higher in the SOD and P1 zeolites, at 80 and 70%, respectively. Iron appears in two crystalline forms, as a sextet for α-Fe^0^ (bcc) and a singlet for γ-Fe^0^ (fcc), with varying ratios. In the SOD and P1 samples, a second sextet (Sx2) was observed, characterized by broader lines and a lower internal magnetic field, indicating the presence of another metal in close proximity to iron, probably nickel, e.g., in the trevorite phase [42].

**Figure 8:**
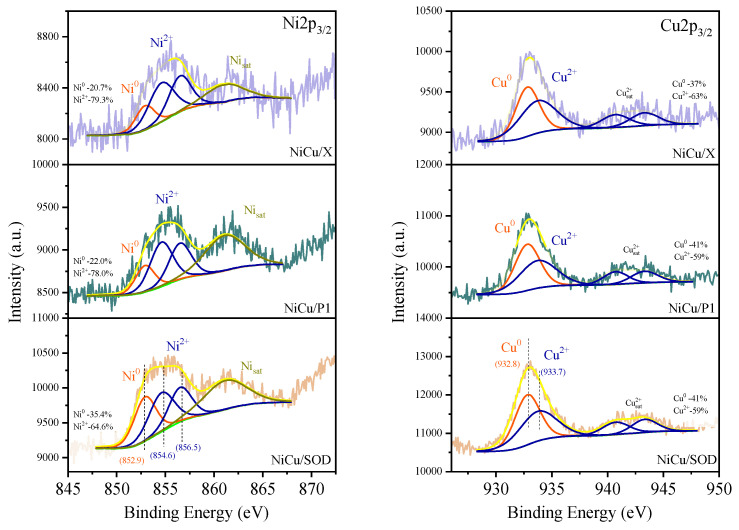
XPS spectra of the metal-modified zeolites.

Figure 8 presents the XPS spectra of the reduced NiCu/P1, NiCu/SOD, and NiCu/X catalysts. The surface composition, based on the XPS spectra, is shown in Table 6. The Ni 2p_3/2_ spectra of all the samples display peaks at 854.6 eV and 856.5 eV, corresponding to Ni^2^⁺, whereas the peak at 852.6 eV is characteristic of Ni^0^ [43]. By deconvoluting the Cu2p_3/2_ spectra, the peak at 932.8 eV can be attributed to metallic copper, whereas the peak at 933.7 eV and the accompanying satellites indicate the presence of Cu^2^⁺ ions [44]. CuLMM Auger spectra (Figure S1) confirm the presence of Cu^0^ and Cu^2+^ species. The appearance of oxidized metals forms is probably due to the spontaneous surface oxidation of the catalysts in the air before testing. The XPS results clearly show that both Ni and Cu were deposited on the surface of the catalysts. The highest surface nickel and copper contents were found on the NiCu/SOD zeolite, compared to the other two catalysts. Its surface nickel content was lower than expected from the bulk composition (~5.5 at. %), but the copper content was close to its nominal value (~2.5 at. %). The Ni/Cu surface ratio was far from the nominal ratio of ~2, instead varying between 1.2 and 1.4 for all the samples. The P1 and X samples contained significantly lower amounts of Cu and Ni on the surface, compared to the SOD catalyst. The iron content for all the samples ranged from 1.2 to 1.8 at. %, which is significantly lower than the nominal value of 8 wt.%. The XPS results further confirm that most of the iron remains below the surface, most probably in the ionic position of the zeolite lattice or in the form of an iron silicate. These findings contrast with our former experience with zeolite X, loaded with a 10 wt.% of Cu and Ni [45], where nickel surface enrichment was found. The Mössbauer spectroscopic results confirmed the formation of a nickel iron alloy in all three samples, so probably a part of the nickel became inactive from a catalytic point of view.

The catalytic activity of the bimetallic zeolites was studied in hydrodeoxygenation of LA to γ-valerolactone at 150 °C and 200 °C reaction temperatures, and the results are presented in Table 7. The main detected products were GVL and 4-hydroxy pentanoic acid (4-HPA). The NiCu/X and SOD zeolites exhibited similar, high activity of around 70% at 150 °C. The selectivity to GVL was also high, at 82%. The NiCu/P1 catalyst, however, showed much lower activity at 16%, although it maintained a high selectivity to GVL (87.5%). The higher reaction temperature, 200 °C, resulted in the total conversion of LA on NiCu/X and NiCu/SOD and in 76–77% selectivity to GVL. A significant increase of LA conversion was also detected on NiCu/P1 (46%), with higher selectivity to GVL (91%) at the higher reaction temperature. The reaction proceeds through the hydrogenation of the ketone group of LA, leading to the formation of 4-hydroxyvaleric acid, which then undergoes dehydration followed by intramolecular esterification to form GVL formation. Thermodynamically, this hydrogenation pathway of LA to GVL is favored and is dominant at low temperatures [46]. Hydrogen is activated on metallic sites, while the dehydration step is driven by the acidic function of the catalyst. The concentration, the strength, and the balance between the two types of active sites determine the catalyst’s performance and product content. The reaction pathway can be influenced by properly adjusting the distribution of active sites and varying the reaction conditions (e.g., gas phase or liquid phase reactions). Metallic functionalities play a key role in the dissociative adsorption of hydrogen. In this regard, the presence of intermetallic or alloy phases can influence the adsorption, such as, in our case, by the formation of the NiCu compound on all the catalysts [47]. Yang et al. reported [48] that metallic nickel in vicinity to partially reduced metal oxide species had a favorable effect on LA adsorption due to the formation of a heterojunction in the band gap. Metallic nickel is responsible for H_2_ adsorption and the subsequent dissociation into hydrogen atoms. This synergy leads to high catalytic activity and GVL selectivity. The presence of active sites that play complementary roles is crucial for efficient GVL production. The strong hydrogenation activity of nickel can be modulated by diluting it with a second metal, such as copper. Copper has a lower hydrogenation activity and tends to occupy the most active, surface defect sites of nickel nanoparticles. Because of the lower hydrogenation activity, the reaction becomes more selective and stops at the formation of GVL, and the consecutive step to pentanoic acid is hindered. The latter was supported by the surface Ni/Cu ratio being between 1.2 and 1.4 of the catalysts detected by XPS (Table 6). The formation trevorite phase in P1 and SOD, as it was detected by the Mössbauer and XRPD methods, could also have positively influenced the selectivity to GVL.

The zeolite support provides the necessary acidic function. In zeolites, Brønsted and Lewis acid centers can be mutually present. CFAZs usually have a high metal content as a contaminant, which compensates the Brønsted acid sites in the lattice, such as AlO^+^, Na, Fe, Ca, and Mg, irrespective of the Si/Al ratio. However, these cationic sites can also serve as Lewis acid centers, as demonstrated in our previous studies using the FT-IR spectroscopy of adsorbed pyridine on Na-X fly ash zeolite [49]. The strength of these acid sites is also critical, with a moderate to high strength being preferred [49]. Our previous study also revealed that Lewis centers in amorphous mixed oxide, formed through zeolite destruction, are somewhat stronger than those in the zeolitic lattice. Nevertheless, the Lewis acid sites of CFAZs are not strong enough to direct the reaction toward the angelica lactone formation, as experienced, in most cases, on H-form zeolites or on γ-alumina supports.

The review of the literature revealed that there is no direct correlation between the hydrogenation performance and specific surface area of the catalysts. For instance, NiCuX and NiCu/SOD catalysts have very different specific surface areas, 100 and 27 m^2^/g, respectively, but their catalytic activity is nearly identical at both reaction temperatures. Similarly to SOD, P1 has also a lower surface area, but the activity is significantly decreased, most probably due to the inaccessibility of metallic phases. The XRPD pattern of spent NiCu/P1 catalysts shows a much smaller amount of metal phase and the formation of nickel ferrite spinel-type mixed oxide (trevorite). This indicates that impregnated metals react strongly with the different constituents of the support, hindering the formation of zero-valent metals, which is also evidenced by the much lower reducibility of the catalyst.

Using water as a solvent poses a challenge due to the deterioration of the carrier and the leaching effects. Hengst et al. [50] observed a loss in activity for Ni/Al_2_O_3_ catalysts due to the formation of a boehmite phase. In our case, a hydrothermal reaction occurred under the reaction conditions, leading to structural change in the zeolites.

The spent NiCu/X and NiCu/SOD catalysts were regenerated and used in a second reaction cycle (Table 7). The LA conversion remained the same high value of 100%, indicating the preservation of active sites during the catalytic test and the regeneration procedure. Moreover, improved selectivity to GVL was registered for all reused catalysts.

The spent catalysts were studied by XRD and Mössbauer spectroscopy (Figure 1B and Figure S2 and Table S2). Based on the XRD data, the most significant change in the spent catalysts was the amorphization of zeolite phases in the SOD and X zeolites. The disappearance of zeolite reflections and the appearance of a broad halo around the 25°2θ value is a sign of amorphous alumina–silica formation. However, the active metallic phases remained unchanged, and the NiCu intermetallic compound showed the same crystallite size as before the catalytic reaction (Table 2). The copper phase became more dispersed after the reaction in the X and P1 zeolites, while it agglomerated on the SOD zeolite. This observation supports the hypothesis that the formation of an intermetallic compound helps stabilize the metallic phases and prevents agglomeration. A partially oxidized form of copper (Cu_2_O, cuprite) also appeared on the spent catalysts with large crystallite sizes exceeding 120 nm. This phenomenon can be explained by the redispersion of metallic phases during the reaction in the water solvent system and the partial oxidation of the catalysts upon exposure to air.

The Mössbauer spectroscopic results for the used catalyst (Table S2) revealed some oxidation of the iron content during the reaction, as evidenced by the disappearance of sextets characteristic of zero-valent iron and the increased amount of Fe^3+^ and Fe^2+^ (Db1 and Db2) species. Additionally, new sextets appeared, with parameters typical of magnetite (Fe_3_O_4_, Sx1 and Sx2). These results support the rearrangement of metallic species and the partial oxidation of them during the catalytic run, consistent with the XRPD patterns of spent catalysts.

**Figure 9:**
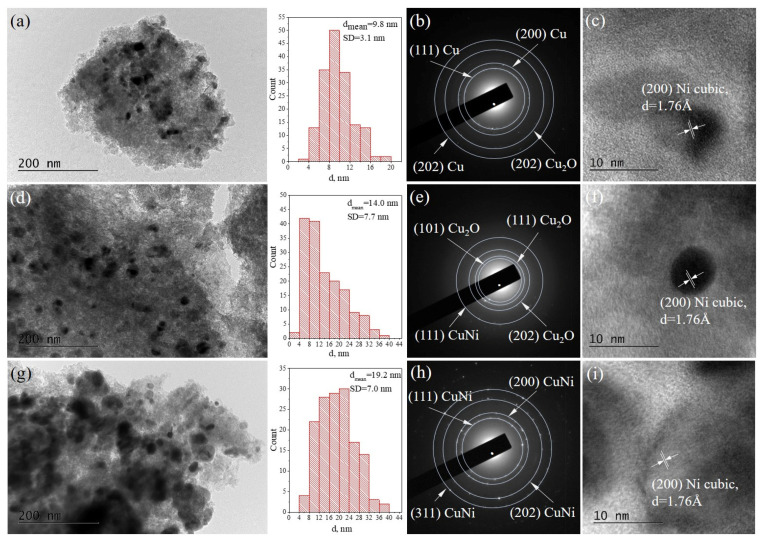
Bright Field TEM micrographs, the size distribution of the metal/alloy nanoparticles, and the corresponding SAED patterns and HRTEM images of the spent samples: (**a**–**c**) spent NiCu/X, (**d**–**f**) spent NiCu/P1, and (**g**–**i**) spent NiCu/SOD.

The crystalline phases identified by the TEM analysis of the spent catalysts (Figure 9) are very similar to the parent-reduced samples and consist with Cu, Cu_2_O, metallic Ni, and intermetallic NiCu alloys. The phases, identified by TEM, are in accordance with the data received from the XRD of the spent catalysts. The histograms reveal that the nanoparticle size distributions do not differ drastically after the reaction and the mean particle diameters remain unchanged in the frame of the standard deviation. In addition, the mean diameters coincide well with those based on the XRD analysis. The results are presented in Table 2.

The XPS spectra of the spent catalysts are presented in Figure S3. The Ni 2p_3/2_ spectra of all the spent catalysts display a peak at 856.2 eV, whereas the peak at 852.8 eV, characteristic of Ni^0^, was detected only for the spent NiCu/SOD and NiCu/X samples. The former peak at 856.2 eV is assigned to the presence of Ni^2+^ species, differing from the fresh catalysts, probably associated with the formation of the NiFe_2_O_4_ phase, which is hardly reducible [43]. The Cu2p_3/2_ spectra indicate the presence of Cu^0^ and Cu^2+^ species [44]. The XPS results confirm that Ni and Cu were still deposited on the surface of the spent catalysts. However, the changes in their state compared to the initial catalysts indicate that a part of them was transformed to hardly reducible NiO species.

## 3. Experimental Section

### 3.1. Materials

The reagent used for the preparation of the coal fly ash zeolite was sodium hydroxide (NaOH, Sigma, St. Louis, MO, USA). Levulinic acid (98%, Aldrich) was used without further purification. The modification of the zeolite was performed with copper nitrate (Cu(NO_3_)_2_·6H_2_O, Sigma-Aldrich, Burlington, MA, USA) and nickel nitrate (Ni(NO_3_)_2_·6H_2_O, Sigma-Aldrich, Burlington, MA, USA).

All the chemicals were used as received without any purification.

### 3.2. Synthesis of Coal Fly Ash Zeolite

The coal fly ash used as a raw material for the synthesis of the zeolites was sampled from two large coal-fired power plants in the Republic of Bulgaria, TPP “AES Galabovo” and TPP “Maritsa 3 Dimitrovgrad”. Both TPPs burn lignite coal, but TPP “AES Galabovo” (AES) is supplied by coal from the “East Maritza” coalfield, which has a low content of limestone, while TPP “Maritsa 3 Dimitrovgrad” (DM) is fed with coal mixtures from the “East Maritza” and “West Maritza” coal deposits, which have a low and a high content of limestone, respectively. The coal fly ash (CFA) samples from the electrostatic precipitators of the two TPPs have been thoroughly investigated in terms of their morphology, chemical, and phase composition in our previous publications [36,51] and are categorized as Class F, according to the international CFA classification standard ASTM 618, as both contain SiO_2_ + Al_2_O_3_ + Fe_2_O_3_ ≥ 70 wt.% [52]. However, the differences in the starting composition of the coal are reflected in the macrocomponent chemical composition of the ash residue from the two combustion plants, concerning mainly the lime and iron oxide content, and CFA from TPP “AES Galabovo”, denoted further as CFA_AES_, contains 4.45 wt.% CaO and 12.99 wt.% Fe_2_O_3_, while CFA from TPP “Maritsa 3 Dimitrovgrad”, referred to hereafter CFA_DM_, comprises 9.36 wt.% CaO and 4.68 wt.% Fe_2_O_3_. By other CFA classification standards [53], CFA_DM_ could be assigned to the medium-calcium Class CI. In our previous studies, both calcium oxide and iron oxides were found to be transferred into the composition of the zeolite products of CFA and have effects on the textural characteristics, adsorption, and catalytic activity of CFAZs [49,51]. Both coal ash residues, CFA_AES_ and CFA_DM_, are consist with individual micron-sized particles, but with a significantly different morphology, as spherical particles are found in CFA_DM_, typical of ashes with increased calcium contents, while CFA_AES_ is composed of agglomerates of various shapes [51]. The two ash residues are similar in their phase composition, with quartz, mullite, magnetite, hematite, and portlandite phases being observed [51]. In our previous studies, both CFAs were successfully applied as raw materials for obtaining nanocrystalline zeolite Na-X [36], investigated as catalysts for low-thermal VOCs oxidation and carbon capture applications [49,51]. Zeolite X crystallizes as a metastable phase at mild conditions, and the thermodynamical order observed during zeolite syntheses investigations reveals opportunities to obtain other zeolite phases from the same starting materials by varying the main hydrothermal synthesis parameters: reaction slurry alkalinity, temperature, and the duration of the hydrothermal treatment [35]. Mixtures of coal ash and alkaline activator sodium hydroxide (NaOH) in a weight ratio of 5 CFA/8 NaOH were dissolved in distilled water until the alkalinity of the suspension was 2 mol/L; they were homogenized with ultrasonic treatment and, after aging for 24 h, they were subjected to hydrothermal synthesis in reactors of stainless steel with Teflon inner pots. After the synthesis, the powdered products were removed by filtration, washed with distilled water to a neutral pH, and, after drying at 105 °C, were subjected to subsequent studies. In the two-step synthesis, the solid-phase reaction mixtures were fused in nickel crucibles for 1 h at 550 °C; after cooling, the resulting fused changes were ground and dissolved in distilled water, ultrasonically homogenized, and, after aging the suspensions for 24 h at room conditions, were subjected to hydrothermal synthesis, as described in the above procedure. In some of the syntheses, to preserve the alkalinity but increase the concentration of Na^+^-ions in the reaction suspensions, a neutral sodium salt, NaCl, was added. The conditions of the conducted syntheses are summarized in Table 1.

### 3.3. Impregnation with Ni and Cu Nanoparticles of Nanosized Coal Fly Ash Zeolite

An incipient wetness impregnation technique was used with nickel and copper nitrates to load 5 wt.% and 2.5 wt.% of the metals, respectively. The support was dried at 160 °C for 2 h prior to impregnation. The bimetallic NiCu samples were prepared by dissolving 0.2608 g Ni(NO_3_)_2_·6H_2_O and 0.0975 g Cu(NO_3_)_2_·3H_2_O in 2 mL distilled water, which was then added simultaneously to 1 g of each support material. The samples were homogenized and dried at room temperature for 24 h. The metal salts were decomposed by calcination at 400 °C for 4 h, with a 3 °C-per-minute heating rate. The catalysts were denoted as NiCu/SOD, NiCu/P1, and NiCu/X.

### 3.4. Characterization

X-ray powder diffraction patterns were recorded by a Bruker D8 Advance diffractometer (Bruker AXS, Karlsruhe, Germany) with Cu Kα radiation and a LynxEye detector between 5 and 80°2θ, with a constant step of 0.02°2θ. The crystallite sizes of the metal oxides were determined by the Sherrer equation, evaluating the FWMH values of the oxide phases with the full profile fitting method. For the identification of the zeolite and metal/metal oxide phases, the following cards were used from the ICDD PDF2 database: NaX: 00-072-2422; NaSOD: 00-037-0476; NaP1: 00-039-0219; Phillipsite: 00-047-0751; Ni^0^: 00-004-0852; Cu^0^: 00-004-0836; Ni_x_Cu_y_: 00-066-0202; and NiFe_2_O_4_: 00-044-0485.

The specific surface area and the pore volume of the samples were determined from N_2_ physisorption isotherms, collected at −196 °C using AUTOSORB iQ-C-MP-AG-AG (Quantachrome Instruments, Anton Paar brand, Boynton Beach, FL, USA). The samples were pretreated at 350 °C in a vacuum before nitrogen adsorption. The total pore volume was determined according to the Gurvich rule at 0.95 relative pressure.

The temperature-programmed, reduction-thermogravimetric analysis (TPR-TGA) investigations were performed by a STA449F5 Jupiter type instrument of NETZSCH Gerätebau GmbH (Netzsch, Waldkraiburg, Germany). In a typical measurement, 20 mg of sample was placed in a microbalance crucible and heated in a flow of 5 vol.% H_2_ in Ar (100 cm^3^/min) up to 500 °C at a rate of 5 °C/min, with a final hold-up of 1 h. Prior to the TPR experiments, the samples were treated in situ at 500 °C in an air flow (10 °C/min) for 1 h.

The Mössbauer spectra were recorded by a Wissel (Wissenschaftliche Elektronik GmbH, Starnberg, Germany) electromechanical spectrometer, working in a constant acceleration mode. A ^57^Co/Rh (activity ≈ 10 mCi) source and α Fe standard were used. The spectra were fitted using software WinNormos-for-Igor6 (Version 6.0).

The surface composition and electronic structure of the materials were investigated by X-ray photoelectron spectroscopy (XPS). The measurements were performed in an ESCALAB Mk II (VG Scientific, Manchester, UK) system using AlK α radiation with an energy of 1486.6 eV. The energy calibration was performed by normalizing the C1s line of the adsorbed adventitious hydrocarbons to 285.0 eV. The binding energies (BE) were determined with an accuracy of ±0.1 eV. XPSPEAK41 software was used to deconvolute the XPS data. The element concentrations were evaluated based on the integrated peak areas of C1s, O1s, Ni2p 3/2, Cu2p 3/2, Fe2p, Al2p, Si2p, Ca2p, Na1s, and Mg1s photoelectron peaks after a Shirley-type linear background subtraction using theoretical Scofield’s photoionization cross-sections.

Transmission electron microscopy (TEM) was carried out with a JEOL JEM 2100 (JEOL Ltd., Tokyo, Japan) transmission electron microscope, with a 200 kV accelerating voltage. The powder samples of the three catalysts were pre-suspended in ethanol, sonicated for 3 min, and a micro-quantity of each suspension was dropped onto a standard TEM grid. For the phase identification, the Match software, version 3.13 (Crystal Impact, Bonn, Germany), and the Crystallography Open Database (COD) were used.

### 3.5. Catalytic Experiments

Prior to the catalytic tests, the samples were pretreated for 2 h in a 50 mL-per-minute Ar/H_2_ flow (5% hydrogen) at 550 °C. In a typical catalytic experiment, the 100 mL stainless-steel reactor (Büchi AG, Flawil, Switzerland) was charged with 20 mL of 0.2 M aqueous solution of levulinic acid (Sigma-Aldrich, 98%) and 0.2 g of powder catalyst. The reactor was fed with 30 bars of hydrogen, heated under stirring at 600 rpm to the reaction temperature of 150 °C, and the reaction proceeded for 4 h. The thermocouple was positioned in the reaction mixture for the accurate measurement of the reaction temperature. Samples were analyzed using HP-GC with a Shimadzu column (Kyoto, Japan), cat. Nº221-75940-30 Phase: SH-Rxi; 5MS; Size L30m.

## 4. Conclusions

Bimetallic (5 wt.% Ni and 2.5 wt.% Cu) catalysts were prepared by impregnating different types of coal fly ash zeolite supports by the incipient wetness impregnation method, such as SOD, X, and P1. The three catalysts exhibited distinct textural properties and hydrogenation/acidic functionalities. Catalytic studies on the hydrodeoxygenation of lignocellulosic biomass-derived levulinic acid to GVL revealed that the formation of Ni_x_Cu_y_ (x = 0.67) intermetallic nanoparticles significantly influenced the catalytic performance. The cationic position of the zeolite support is occupied by various cations, such as Fe, Na, Ca, Ni, and Cu, and, therefore, they show a weak Lewis acidic character. Consequently, the dehydration activity of the catalysts is hindered, and, instead, the hydrogenation activity is the rate-determining step of the reaction. However, the strong hydrogenation character of nickel is suppressed by the formation of the NiCu intermetallic phase, avoiding the further hydrogenation of γ-valerolactone to pentanoic acid and, thus, making the reaction more selective. It turned out that the textural parameters have a less pronounced effect on catalytic activity than the dispersion of metals, and even the collapse of the zeolite structure did not negatively affect it. The CuNi/SOD and CuNi/X catalysts showed similar catalytic behaviors: total LA conversion and a high GVL yield (76–77%) was detected at a reaction temperature of 200 °C. The P1 zeolite had less favorable properties as a support: its structural changes during the preparation procedure and the catalytic run decrease the accessibility of catalytically active metal species.

## Figures and Tables

**Table 1:** Parameters of composition, fusion, ultrasonic, and hydrothermal treatments during the synthesis of the CFAZ samples.

Samples	CFA Source	NaOH/ CFA/NaCl	Fusion Temp. °C	U/S Time min	HT Temp. °C	HT Time h
F0	AES	5/10	550	15	90	4
H0	AES	5/10	-	30	90	4
F1	AES	5/8	550	15	140	12
F2	AES	5/8	550	15	120	24
F3	AES	5/8	550	15	160	6
F4	AES	5/8	550	15	120	36
F5	AES	5/8/2	550	15	160	8
F6	DM	5/8	550	15	160	6
F7	DM	5/8	550	15	140	12
F8	DM	5/8	550	15	120	24
H1	AES	5/8	-	30	140	12
H2	AES	5/8	-	30	120	24
H3	AES	5/8	-	30	160	6
H4	AES	5/8	-	30	120	36
H5	AES	5/8/2	-	30	160	8
H6	DM	5/8	-	30	160	6
H7	DM	5/8	-	30	140	12
H8	DM	5/8	-	30	120	24

HT—hydrothermal treatment, U/S—ultrasonic treatment.

**Table 2:** Crystalline phases of the studied catalysts, determined by the XRPD method.

Samples	Zeolite Type	Crystallite Size (nm)		
		Ni_x_Cu_1−x_ *	Cu^0^	Cu_2_O
NiCu/SOD	SOD	21	43	-
Spent cat.	-	23	124	124
NiCu/X	NaX	13	123	
Spent cat.	-	12	26	>1000
NiCu/P1	P1	13	26	-
Spent cat.		13	26	123

* a_0_ = 3.554, x = 0.67.

**Table 3:** Textural data of the samples studied.

Samples	S_BET_ m^2^/g	S_micro_ m^2^/g	V_micro_ cm^3^/g	V_tot_ cm^3^/g
SOD	52	-	-	0.091
NiCu/SOD	27	-	-	0.063
P1	41	0.5	0.001	0.070
NiCu/P1	21	-	-	0.053
X	380	266.8	0.110	0.261
NiCu/X	100	46.8	0.020	0.119

V_tot_—Total pore volume, calculated at 0.95 relative pressure.

**Table 4:** Mössbauer parameters of the initial CFAZs.

Samples	Components	*δ* mm/s	Δ (*2ε*) mm/s	*B_hf_* T	*Γ_exp_* mm/s	G %
X	Db, Fe^3+^	0.29	0.76	-	0.52	100
SOD	Db, Fe^3+^	0.26	0.68	-	0.54	56
	Sx, Fe^3+^	0.26	−0.01	46.2	2.02	44
P1	Db, Fe^3+^	0.33	0.96	-	0.76	39
	Sx1, α-Fe_2_O_3_, Fe^3+^	0.37	−0.20	51.4	0.28	10
	Sx2, Fe_3_O_4_, Fe^3+^_tetra_	0.34	0.00	48.3	0.49	18
	Sx3, Fe_3_O_4_, Fe^2.5+^_octa_	0.58	−0.05	44.5	0.58	33

**Table 5:** Mössbauer parameters of the studied metal-modified catalysts.

Sample	Components	*δ* mm/s	Δ (2*ε*) mm/s	*B_hf_* T	*Γ_exp_* mm/s	G %
NiCu/X	Sn, γ-(Fe,Ni) alloy, Fe^0^	−0.08	-	-	0.32	13
	Db1, Fe^3+^	0.27	0.92	-	0.90	30
	Db2, Fe^2+^	0.95	2.18	-	1.20	21
	Sx, α-(Fe,Ni) alloy, Fe^0^	−0.03	0.01	32.7	0.44	36
NiCu/SOD	Sn, γ-(Fe,Ni) alloy, Fe^0^	−0.02	-	-	0.49	15
	Sx1, α-(Fe,Ni) alloy, Fe^0^	−0.04	0.02	32.8	0.33	31
	Sx2, α-(Fe,Ni) alloy, Fe^0^	0.00	0.10	26.8	1.00	37
	Db, Fe^3+^	0.28	0.97	-	0.60	17
NiCu/P1	Sn, γ-(Fe,Ni) alloy, Fe^0^	−0.04	-	-	0.40	4
	Db1, Fe^3+^	0.32	0.89	-	0.90	20
	Db2, Fe^2+^	0.95	1.79	-	0.87	11
	Sx1, α-(Fe,Ni) alloy, Fe^0^	0.00	0.00	32.8	0.29	36
	Sx2, α-(Fe,Ni) alloy, Fe^0^	0.03	0.00	29.0	1.79	29

**Table 6:** Surface chemical composition of the samples, based on the XPS data (at. %).

Samples	O	Ni	Cu	Mg	Fe	Al	Si	Ca	Na
NiCu/SOD	49.3	2.8	2.3	0.5	1.5	16.6	17.6	2.7	6.7
NiCu/P1	53.7	2	1.4	1.2	1.2	10.6	20.4	3.1	6.4
NiCu/X	55.6	1.1	0.9	1	1.8	11.3	18.2	3.0	7.1

**Table 7:** The catalytic conversion of LA to GVL on the zeolite-supported NiCu catalysts (reaction parameters: 150/200 °C; 4 h; 30 atm H_2_; LA 4 mmol/20 mL H_2_O; H_2_/LA = 30; water solvent; 0.2 g catalyst).

Samples	Temp. °C	LA Conv. (%)	GVL Yield (%)	HPA Yield (%)
NiCu/X	150	71	59	12
NiCu/P1	150	16	14	2
NiCu/SOD	150	72	59	13
NiCu/X	200	100/100 *	77/91 *	23/9 *
NiCu/P1	200	46	42	4
NiCu/SOD	200	100/100 *	76/90.5 *	24/10.5 *

LA—levulinic acid; GVL—valerolactone; HPA—4 hydroxy pentanoic acid; * catalytic data obtained with spent catalysts that were regenerated and re-reduced.

## Data Availability

Data are contained within the article and Supplementary Materials.

## Supplementary Materials

The following supporting information can be downloaded at: https://www.mdpi.com/article/10.3390/molecules29235753/s1, Figure S1: CuLMM Auger spectra of the studied catalysts; Figure S2: Mössbauer spectra of the reduced samples after catalytic reaction; Figure S3: XPS spectra of the spent catalysts; Table S1: Content of impurities in the obtained zeolites; Table S2: Mössbauer parameters of the spent catalysts.
