# Supplementary material for: The Formation of γ-Valerolactone from Renewable Levulinic Acid over Ni-Cu Fly Ash Zeolite Catalysts
Source: Molecules. 2024 Dec 5;29(23):5753. doi: 10.3390/molecules29235753 (PMC11643475; doi:10.3390/molecules29235753)
Supplement: Supplementary file 1 [file molecules-29-05753-s001.zip › molecules-3306816-supplementary.pdf]

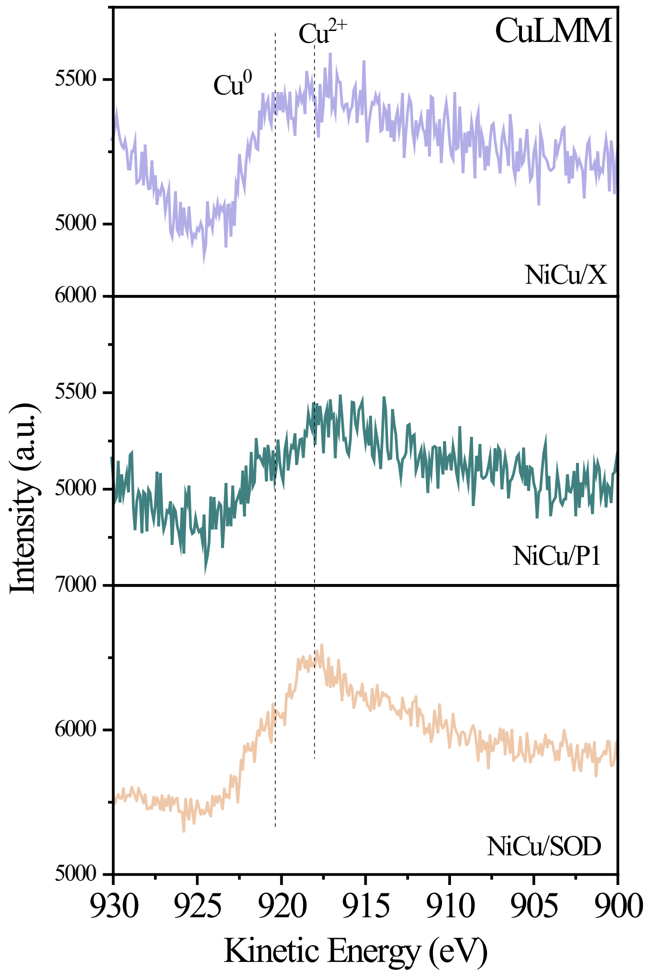

Figure S1. CuLMM Auger spectra of the studied catalysts

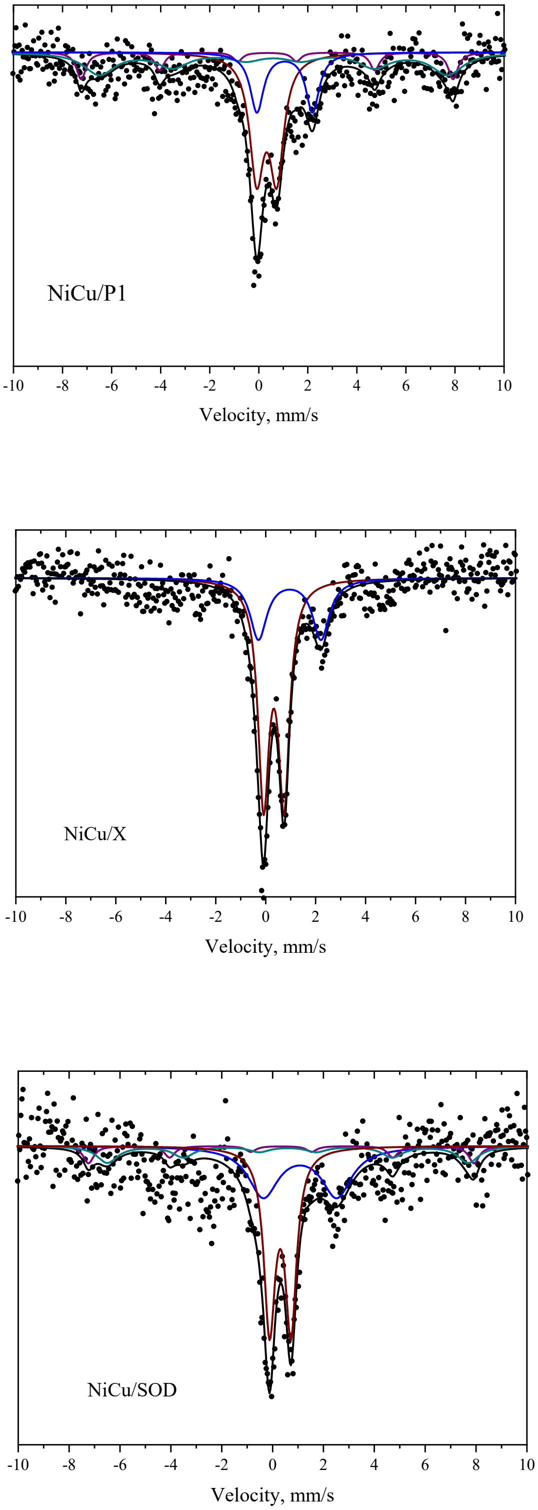

Figure S2. Mössbauer spectra of the reduced samples after catalytic reaction

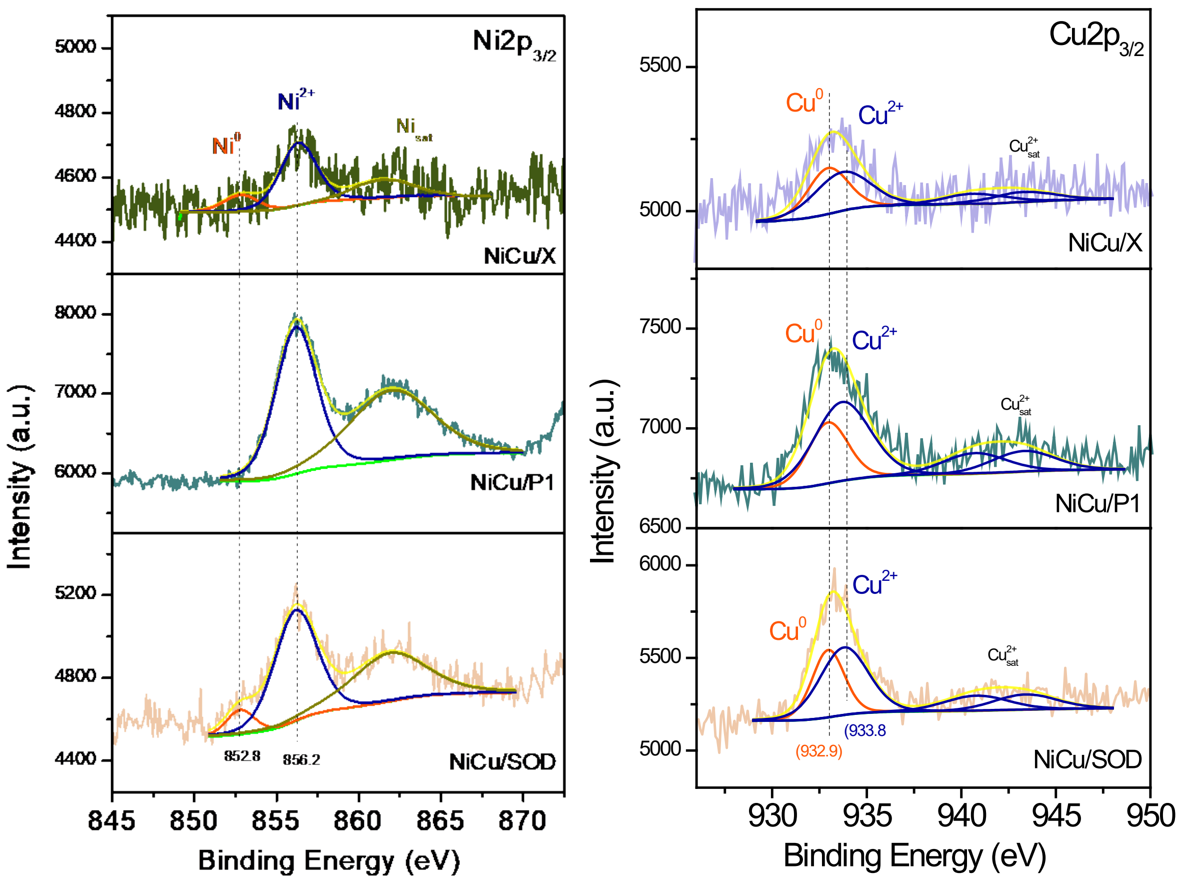

Figure S3. XPS spectra of the spent catalysts

Table S1. Content of impurities in the obtained zeolites.

Impurities	NaX	SOD	P1
Ti, wt.%	0.300	0.400	0.200
Ca, wt.%	3.500	3.600	3.500
Mg, wt.%	1.200	1.300	2.200
Na, wt.%	4.100	6.100	4.500
K, wt.%	0.250	0.240	0.210
Fe, wt.%	7.700	7.800	7.900
Mn, wt.%	0.024	0.053	0.052
Sr, wt.%	0.041	0.042	0.043
S, wt.%	0.060	0.250	0.150
Cu, ppm	154	183	175
Zn, ppm	110	134	142
Cr, ppm	16	55	48
Ni, ppm	245	128	71
Ba, ppm	366	442	436
Li, ppm	23	39	29
B, ppm	77	96	98
Co, ppm	5	6	5
Pb, ppm	<10	<10	10
Cd, ppm	<5	<5	<5
Bi, ppm	<6	<6	<6

Table S2. Mössbauer parameters of the spent catalysts.

Sample	Components	δ, mm/s	Δ (2ε), mm/s	B_{hf}, T	Γ_{exp}, mm/s	G, %
Spent NiCu/X	Db1, Fe^{3+}	0.33	0.82	-	0.54	70
	Db2, Fe^{2+}	0.96	2.50	-	0.82	30
Spent NiCu/SOD	Db1, Fe^{3+}	0.30	0.84	-	0.54	44
	Db2, Fe^{2+}	1.08	2.88	-	1.44	33
	Sx1, Fe_3O_4, Fe^{3+}_{tetra}	0.34	0.00	47.0	0.50	8
	Sx2, Fe_3O_4, $Fe^{2.5+}_{octa}$	0.60	0.00	44.0	1.00	15
Spent NiCu/P1	Db1, Fe^{3+}	0.32	0.82	-	0.70	39
	Db2, Fe^{2+}	1.06	2.27	-	0.67	18
	Sx1, Fe_3O_4, Fe^{3+}_{tetra}	0.34	0.00	47.0	0.47	12
	Sx2, Fe_3O_4, $Fe^{2.5+}_{octa}$	0.60	0.00	44.0	1.54	31
